# Transcription Factor EepR Is Required for *Serratia marcescens* Host Proinflammatory Response by Corneal Epithelial Cells

**DOI:** 10.3390/antibiotics10070770

**Published:** 2021-06-24

**Authors:** Kimberly M. Brothers, Stephen A. K. Harvey, Robert M. Q. Shanks

**Affiliations:** Charles T. Campbell Ophthalmic Microbiology Laboratory, Department of Ophthalmology, University of Pittsburgh School of Medicine, Pittsburgh, PA 15213, USA; kmb227@pitt.edu (K.M.B.); stephenaharvey@cs.com (S.A.K.H.)

**Keywords:** bacterial infection, *Serratia marcescens*, transcription factor, keratitis, ocular surface, epithelium, cornea, metabolomics

## Abstract

Relatively little is known about how the corneal epithelium responds to vision-threatening bacteria from the Enterobacterales order. This study investigates the impact of *Serratia marcescens* on corneal epithelial cell host responses. We also investigate the role of a bacterial transcription factor EepR, which is a positive regulator of *S. marcescens* secretion of cytotoxic proteases and a hemolytic surfactant. We treated transcriptomic and metabolomic analysis of human corneal limbal epithelial cells with wild-type bacterial secretomes. Our results show increased expression of proinflammatory and lipid signaling molecules, while this is greatly altered in *eepR* mutant-treated corneal cells. Together, these data support the model that the *S. marcescens* transcription factor EepR is a key regulator of host-pathogen interactions, and is necessary to induce proinflammatory chemokines, cytokines, and lipids.

## 1. Introduction

The cornea, the transparent, anterior layer of the eye, is essential for vision and protected by numerous host immune factors, the tear film [1,2], and the corneal epithelium [3,4]. When the epithelium is damaged or compromised, it permits entry of microbes into the stroma where they can multiply and cause damage to the ocular tissues; the progression of infection is rapid, sometimes leading to corneal perforation from bacterial proteases and from the ensuing inflammatory response [5,6,7,8,9].

*Serratia marcescens* is a gram negative pathogen from the order Enterobacterales frequently isolated from contact lenses, and associated with ocular infections [10,11,12]. Bacteria are linked with chronic infections, non-healing wounds, and are thought to prevent wound closure; however, the impact of bacteria on corneal infection and wound healing is poorly understood [13,14,15]. Our previous study identified *S. marcescens* LPS as being sufficient to inhibit corneal epithelial wound closure and further identified transposon insertions in genes that rendered the bacterium unable to inhibit corneal cell migration, but the role of these genes in ocular surface host-pathogen interactions was not characterized [16]. One mutation mapped to the *eepR-eepS* locus, that codes for a hybrid two-component transcription factor system involved in virulence factor secretion, cytotoxicity to mammalian cells, and proliferation in a rabbit keratitis model [17,18,19].

Previous studies have evaluated the impact of bacteria on the global transcriptomic response of corneal cells, but this has only been done with *Pseudomonas aeruginosa* and *Staphylococcus aureus* [20,21,22,23]. In this study, the role of the EepR transcriptional regulator in the corneal epithelial cell transcriptional and small molecule response to *S. marcescens* was evaluated. We report that in contrast to other pathogens, mutation of one bacterial transcription factor in *S. marcescens* had a broad impact on epithelial cell responses, including reduced expression of inflammatory markers and lipid metabolism genes.

## 2. Results

### 2.1. HCLE Cells Exposed to eepR Mutant S. marcescens Secretomes Have an Attenuated Inflammatory Response Compared with Wild-type Treated HCLE Cells

To increase our understanding of the corneal response to an order of bacteria not previously tested, a global transcriptional analysis of the HCLE cells was performed. Here we used a wild-type (WT), low cytotoxicity [24] isolate of *S. marcescens* (PIC3611), and an isogenic strain with a deletion in the *eepR* gene that was previously described [19] to further investigate EepR’s role in how bacteria influence corneal biology. In this study, bacterial secretomes were used to stimulate corneal cells because we have previously shown wild-type secretomes to strongly influence the behavior of a human corneal epithelial cell line and because secretomes are less toxic to corneal cells [16,25,26]. Confluent monolayers of the human corneal limbal epithelial (HCLE) cell line were first exposed to *S. marcescens* WT secretome for 0, 1, 2, 3, 4, and 5 h to determine the time frame for maximal stimulation by assessing levels of the cytokine TNFα. The 5 h exposure time point was chosen based upon our preliminary findings (data not shown) and from a previous ELISA-based study of human corneal epithelial cell inflammatory response to *S. marcescens* [27].

Next, we compared the transcriptomes of mock-treated (LB medium in equal volume as secretomes) corneal cells with those exposed to normalized secretomes from WT or *eepR* cells. Lower case *eepR* refers to the mutant strain. As noted in Materials and Methods, 21,932 microarray panels (unique target sequences) yielded reliable data; valid changes between WT secretome-treated and mock-treated cells occurred in only 2510 panels (11.4%), and of those, only 915 (4.2%) were modulated by 2-fold or more (examples in Table 1 and Table 2). In contrast, valid changes between *eepR* secretome-treated and mock-treated cells occurred in only 798 panels (3.6%), and of those, only 241 (1.1%) were modulated by 2-fold or more (examples in Table 3 and Table 4). Over half of the *eepR* secretome-modulated panels (138, 57%) were present in the WT-treatment group also (see nine genes in common between Table 1 and Table 3, eight genes in common between Table 2 and Table 4), and the direction of modulation was concordant between treatments for all these panels except SPRY2, which was increased by WT treatment and decreased by *eepR*. Visual inspection showed that within this group of 138 genes, whatever the direction of change caused by *eepR* (increase or decrease), its magnitude was always less than that caused by WT. However, some genes outside this group showed greater modulation by *eepR* than by WT. Accordingly, the scaled *eepR* response (*eepR* − control)/|(WT − control)| was also calculated (Table 5 and Table 6).

The 915 panels modulated by WT were submitted to Ingenuity Pathway Analysis software (Qiagen, Germantown, MD, USA), yielding 24 significantly enriched (*p* < 0.05) canonical pathways which had adequate z-scores (|z| > 2; see Table 7). At least nine of these pathways address direct or indirect immune functions. When submitted for analysis separately, the 798 *eepR* modulated panels only yielded three significantly enriched pathways, two of which were also WT-modulated (see Table 7). The third pathway (GNRH Signaling) was not significantly enriched by WT treatment. In *S. marcescens* WT secretome-treated HCLEs versus mock-treated cells, the twenty-five most upregulated genes (9.1- to 56.6-fold increase) included genes involved in inflammatory signaling pathways (Table 1). Genes with the greatest decrease (4.9- to 50-fold decrease) in WT secretome-treated HCLEs were those involved in nucleosome assembly, phospholipid metabolic processes, and transcription (Table 2). Moreover, HCLEs-treated with *eepR* secretome showed decreased upregulation of genes for proinflammatory factors; however, genes involved in cell to cell adhesion, leukocyte chemotaxis, transport, and signaling were upregulated (Table 3). Genes with the greatest decrease in *eepR versus* mock-treated secretomes were those involved in nucleic acid binding, transport, and transcription (Table 4).

Interestingly, when we examined our results in the context of scaled *eepR* (*eepR*—control/WT—control), there were also several genes where the expression difference was greater than 10-fold in *eepR*-treated cells in comparison to WT. In particular, there were differences in genes involved in intracellular protein transport, protein binding, transcription, nucleic acid binding, and translation (Table 5). Genes with the lowest expression in the scaled *eepR* response were involved in protein complex assembly, protein binding, signal transduction, actin filament polymerization, inactivation of MAPK activity, and negative regulation of transcription (Table 6).

**Figure 1:**
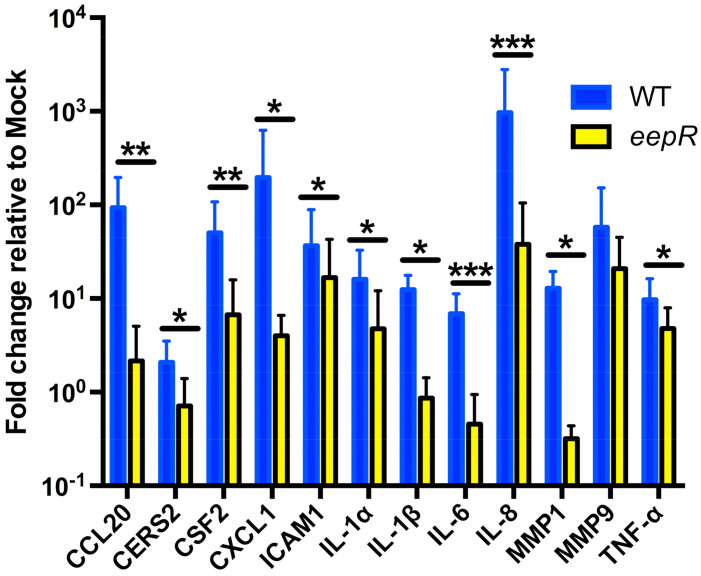
qRT-PCR of pathway markers confirmed microarray analysis. Graph represents the fold change in gene expression relative to mock (LB) treatment. HCLE cells were exposed to LB, WT, and *eepR* transcriptomes of 5 h. Gene expression was normalized to GAPDH expression. Means (n = 4–8, n = 3 for IL-1α) and SD are shown. ∆∆CT values were compared by ANOVA with Bonferroni’s post-test, one asterisk (*) indicate *p* < 0.05, two indicate (**) *p* < 0.01, and three (***) indicate *p* < 0.001.

From our microarray results, we chose genes to validate by qRT-PCR that are known mediators of response to infection and corneal wound healing, involved in cellular signaling, motility, actin binding, and cellular division/membrane organization, and had at least a 2-fold difference when comparing WT to *eepR*-treated HCLEs [17,20,21,27]. Overall, our qRT-PCR results validated changes observed with the microarray, including that the *eepR*-treated HCLEs in most cases had a lower fold change in proinflammatory gene expression (Figure 1, Table 1). We note that, when assayed by qRT-PCR, nine out of the twelve genes show a greater response to WT treatment than they do by microarray analysis, consistent with the greater sensitivity and wider dynamic range of qRT-PCR.

### 2.2. Bacterial Secretomes Influence Corneal Epithelial CellLipid Metabolism

In addition to evidence of EepR playing a role in producing inflammatory markers, microarray analysis revealed alterations in pathways associated with lipid metabolism and signaling. These pathways include ceramide biosynthesis, ceramide signaling, and Sphingosine-1-phosphate receptor signaling with avalid increases in CERS2 (1.5-fold), S1PR3 (2.3-fold), SPHK1 (1.8-fold), and SPTLC2 (2.5-fold) genes by cells treated with wild-type, but not *eepR* secretomes. Increased CERS2 expression observed in the microarray was confirmed by qRT-PCR (Figure 1).

**Figure 2:**
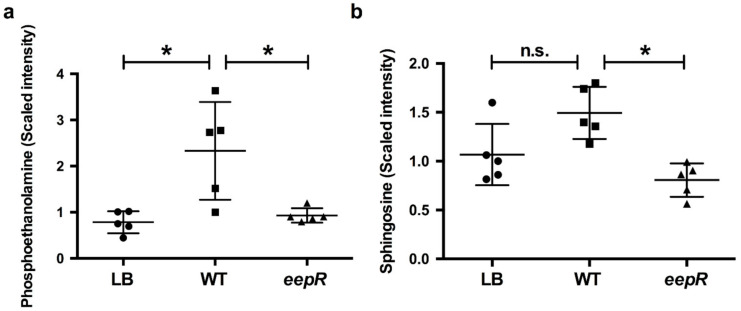
Metabolomic analysis demonstrates alteration of sphingosine and lipid metabolism in corneal cells challenged by *S. marcescens* secretomes. HCLE cells were treated with LB, WT, or *eepR* secretomes for 24 h. Mean and SD (n = 5) of relative amounts of (**a**) phosphoethanolamine and (**b**) sphingosine. Circles = LB (mock treatment), squares = WT, and triangles = *eepR* mutant treated HCLE cells. Asterisks (*) indicate *p* < 0.05 by one-way ANOVA with Tukey’s post-hoc analysis. n.s., not significant.

To further verify the alteration in producing compounds associated with the lipid pathways implicated in the microarray data and to gain insight into the corneal epithelial cell response to enteric bacteria, small molecule metabolomic analysis was performed on HCLE cells exposed to secretomes derived from WT and the *eepR* mutant. Consistent with the results of the microarray analysis and qRT-PCR, the metabolomic analysis identified changes in markers involved in lipid metabolism (Figure 2, Supplementary Tables S1 and S2). There were significant increases in metabolites for lipid metabolism for *S. marcescens* WT-treated HCLEs, including sphingosine, phosphoethanolamine (Figure 2), as well as linoleate, eicosapentaenoate, docosapentaenoate, docosahexaenoate, and myristate (Table S1). Together, these data indicate that *S. marcescens* secreted factors have a major impact on human corneal cells, including increased expression of inflammatory and lipid metabolism pathways, and that *S. marcescens* requires EepR for these effects.

## 3. Discussion

*S. marcescens* EepR, a master transcriptional regulator of secreted enzymes and secondary metabolites, plays an important role in hemolysis, pigment production, swarming motility, and contributes to bacterial proliferation in the cornea. A previous study demonstrated the importance of the *S. marcescens* transcription factor EepR in the regulation of protease production, corneal cell-induced cytotoxicity, and its ability to induce the proinflammatory cytokine IL-1β [17]. Because of its involvement in ocular host-pathogen response, we sought to determine differences in gene expression profiles in *eepR*-treated corneal cells in comparison to WT. Interestingly, genes with the greatest expression in *eepR* mutant-treated corneal cells compared to WT-treated cells were those involved in intracellular transport, protein binding, cellular component movement, cell adhesion, and membrane-related functions (Table 5), suggesting deletion of EepR promotes cell migration and wound healing. Consistently, *eepR*-treated cells were found to regulate lipid metabolic process, transcription, and intracellular protein transport (Table 5) and activate the MAPK pathway, which has been demonstrated to promote cell migration [28]. In contrast, WT-treated cells were found to inactivate the MAPK pathway (Table 6), which is consistent with its wound inhibitory phenotype [16].

The effect of bacteria on human corneal epithelial cells is of interest because bacteria cause the majority of corneal ulcers [29]. A limited number of studies have examined the impact of *P. aeruginosa* and *S. aureus* on the corneal transcriptomic response [20,21,22,23], but these have not been done with bacteria of the Enterobacterales order. Bacteria, such as *Klebsiella*, *Proteus*, and *Serratia*, cause a significant number of ocular infections [30]. There is a unique immunological response of the cornea, being an immune-privileged site. Chidambaram et al. compared gene expression profiles of corneal tissues from microbial keratitis patients infected with *Streptococcus pneumoniae, P. aeruginosa, Fusarium sp.,* and *Aspergillus sp.* to normal corneal tissue from cadavers [20]. In agreement with our own data, they found increased expression of the proinflammatory markers MMP9, MMP1, IL-1β, and TNF with the greatest expression observed in MMP9. In addition to the previously mentioned markers, they also found increases in MMP7, MMP10, MMP12, TLR2, and TLR4, all markers known to promote inflammation and immune recognition [20]. Our data also found a 2.2-fold increase in expression of TLR2 in WT *versus eepR* mutant-treated HCLEs, but no significant changes in TLR4 expression. Microarray gene expression levels for TLR4 were low, but detectable for all conditions in our study. However, expression of TLR4 in corneal epithelial cells has been previously demonstrated to be reduced [31,32], and could explain why our results differed from Chidambaram et al.

The *S. marcescens*-induced proinflammatory gene response reported here was consistent with a study by Hume et al. [27], who used ELISA to explore the cytokine response of human corneal cells and polymorphonuclear monocytes (PMNs) to clinical isolates of *S. marcescens*. Though they found strain differences in cytokine response, there was an overall positive trend in activation of TNFα, IL-6, and CXCL8 after 4 h of exposure to bacteria which was similar to our results after 5 h of exposure [27].

The impact of living *Pseudomonas aeruginosa* upon the transcriptome of murine corneas has been explored by Gao et al. [21]. They reported upregulation of Krt16, MMP10, MMP13, S100A8, Stfna111, and S100A9 genes with an even greater increase in the genes involved in antimicrobial peptide production S100A8 and S100A9, when mice were pretreated with flagellin [21]. Our results were not as striking for S100A8 and S100A9, but did demonstrate a 2-fold increase in WT-treated HCLES in comparison to *eepR*. Huang et al. used murine corneas infected with *P. aeruginosa* and demonstrated upregulation of proinflammatory markers GM-CSF, ICAM1, IL1α, IL-1β, IL-6, TNFα, MMP9, MMP10, and MMP13 in accordance with our results [22]. In addition to the previously mentioned genes, we also observed upregulation of proinflammatory markers CCL20, CERS2, CXCL1, CXCL8, and MMP1.

An elegant study by Heimer et al. used a well-defined reference strain of the gram positive bacteria *S. aureus* to examine corneal epithelial cell responses to bacteria [23]. They evaluated the effect of an isogenic *agr sarA* double mutant of *S. aureus* that has similar defects as our *eepR* mutant in reduced secretion of virulence factors [23]. After treating human corneal cells with *S. aureus*, highly increased expression of proinflammatory markers CCL20, CSF2, CXCL1, IL-6, CXCL8, and TNFα was observed. These results are in agreement with our own, with the only major notable difference being that the gene most induced by *S. marcescens* WT bacteria was CXCL8, a neutrophil chemoattractant important for neutrophil migration to the site of infection and clearance of bacteria, whereas *S. aureus* most induced CCL20 a chemokine with antibacterial properties [33]—the third most highly induced gene in our study. In sharp contrast to their study, while the *S. aureus agr sarA* double mutant caused relatively little change in host response compared to the WT *S. aureus*, the *S. marcescens eepR* mutant was strikingly less able than the WT to induce expression of proinflammatory genes. Another notable difference is that some of the signal transduction factors upregulated by *S. aureus* were not affected by *S. marcescens*, notably the plasminogen activator inhibitor SERPINB2 that is involved in macrophage function and cell migration [34], and the glycoprotein STC1 that is involved in angiogenesis and wound healing [35].

Matrix metalloproteinases (MMPs) are enzymes that function in immune responses to infection in addition to numerous other roles. MMPs are involved in recruiting white blood cells, chemokine and cytokine responses, and cell matrix remodeling [36]. In our study, numerous matrix metalloproteases were upregulated >2-fold by *S. marcescens*, including MMP1, 9, 10, 13, 14, 16, 19, 28, but a similar trend was not described in *S. aureus* challenged cells [23].The different pathogen associated molecular patterns produced by the bacteria and the challenge with whole *S. aureus versus S. marcescens* secretomes (which include flagella and LPS) may account for some of the differences observed. Nevertheless, the *S. marcescens* EepR protein had a much larger role than the *S. aureus* SarA transcription factor and Agr quorum sensing system in affecting the corneal epithelial cell transcriptional response.

The reason for which *eepR* mutants confer such a different transcriptional response compared to the WT is not clear at this time. The *eepR* mutant is defective in the secretion of metalloproteases, such as serralysin and SlpB [17]. Serralysin, also called the 56-kDa protease, was shown in experimental models to have an impact on the immune system, rendering mouse lungs much more susceptible to influenza infection [37]. The protease was shown to increase vascular permeability by activation of the Hageman factor-kallikrein-kinin system [38]. Further studies will evaluate the role of EepR regulated bacterial metalloproteases in corneal wound healing.

Our microarray and qRT-PCR data suggested differences for expression of genes involved in the lipid metabolism pathway for corneal cells exposed to WT, but not *eepR* mutant secretomes. This data was validated using metabolomics approaches and indicated that the changes in transcription yielded measurable differences in the molecules involved in the altered pathways. Bioactive sphingolipids, such as those with altered expression shown here, like ceramide and sphingosine 1-phosphate, are known signaling molecules that mediate wound healing in many tissues [39], and likely play a different role in corneal responses. These data indicate the importance of a single bacterial transcription factor in dictating the corneal cell response as measured through transcriptomic and metabolomic analysis. The findings and their implications should be discussed in the broadest context possible.

## 4. Materials and Methods

### 4.1. Bacterial Growth Conditions and Media

*S. marcescens* cultures were grown in lysogeny broth (LB) [40] at 30 °C with shaking. Bacteria free secretomes of *S. marcescens* WT and *eepR* were prepared by normalizing overnight cultures to OD_600_ = 2.0 and removing the bacteria by centrifugation at 14,000 rpm for two minutes followed by filtration through a 0.22 μm filter.

### 4.2. Microarray

HCLE cell line was obtained from Ilene Gipson [41], and were maintained in KSFM media as previously described [16]. Cells were seeded into 12 well plates at a density of 1.5 × 10^5^ cells per well. Secretomes were prepared as described above and added to HCLE cells at the same dosage (500 μL into 1 mL KSFM) and incubated for 5 h at 37 °C + 5% CO_2_. HCLE cells were washed 3 times with phosphate buffered saline (PBS) and stored in 5 volumes of RNA*later* (Sigma-Aldrich, St. Louis, MO, USA) at 4 °C until used. RNA was extracted with a GenElute Mammalian total RNA miniprep kit (Sigma-Aldrich), treated with 1 unit of RQ1 Dnase (Promega, Madison, WI, USA) for 30 min at 37 °C, and quantified by Nanodrop (Thermo Scientific|Thermo Fisher Scientific, Waltham, MA, USA). 500 ng samples of total RNA were processed using an Affymetrix 3′-IVT Express kit (Affymetrix, Santa Clara, CA, USA) and yielded 43.2 ± 14.4 μg of biotinylated cRNA (mean ± SD, n = 5), with one outlier of 7 μg. Twenty μg of biotinylated cRNA was hybridized to Affymetrix U133 Plus 2.0 GeneChips (catalog #900470). The GeneChips were developed and scanned using an Affymetrix GeneChip 3000 Array Scanner.

The resultant DAT files were consolidated to CEL files, which were analyzed with Affymetrix GCOS v1.4 software, using default parameters. Numerical data and the software flags for Presence/Absence and for significant pairwise changes were transferred to Microsoft Excel. Of the 54,675 panels (unique sequence targets) on the microarray, 26,162 showed no detectable expression in any sample and omitted further consideration. Of the remaining 28,513 panels, the 22,553 (79%) which showed consistent detectable expression in the duplicate samples of at least one experimental group were taken for analysis. Of these, 621 panels (2.8%) showed a significant 2-fold difference between duplicates and were rejected as unreliable. For the reliable 21,932 panels, the ratio (mean (WT − treated)/mean (untreated)) was calculated. This ratio represented a valid change if:Both samples in the higher-expressing group reported Present (i.e., detectable target sequence), all four pairwise comparisons between groups showed significant changes using the GCOS software, and the groups did not overlap.

### 4.3. Quantitative Reverse Transcriptase PCR (qPCR)

RNA was extracted as described above and concentrated using an RNA Clean and Concentration kit (Zymo Research, Irvine, CA, USA). All samples were normalized with nuclease free water to a concentration of 50 ng/µL. 250 µg of RNA was synthesized into cDNA using Superscript III reverse transcriptase (Invitrogen|Thermo Fisher Scientific, Waltham, MA, USA) as previously described [19]. To identify any genomic DNA contamination, non-template controls of each RNA sample were also prepared and verified by reverse transcriptase PCR (RT-PCR) using GAPDH primers [42]. All contaminated samples were discarded. Quantitative reverse transcriptase PCR (qRT-PCR) was performed using Sybr green reagent (Applied Biosystems|Thermo Fisher Scientific, Waltham, MA, USA) using primers for CCL20, CERS2, CSF2, ICAM-1, IL-1α, IL-1β, IL-6, IL-8, MMP1, MMP9, TNFα [42,43,44,45,46,47,48,49,50,51,52]. All gene reactions were normalized to GAPDH [42], and analyzed using the ΔΔCT method. All experiments were performedat least three independent times.

### 4.4. Metabolomics

One sample containing 100 μL of LB (mock) and five 100 μL samples each of WT and *eepR* mutant *were* collected and stored at −80 °C. All samples were collected in two independent harvests on two different days and shipped on dry ice to Metabolon Inc. for small molecule analysis. Samples were prepared using an automated MicroLab STAR^®^ system (The Hamilton Company, Allston, MA, USA) using a proprietary series of organic and aqueous extractions. The prepared extract was then divided into two fractions, one for analysis by liquid chromatography and one for analysis by gas chromatography. Samples were then placed in a TurboVap^®^ (Biotage, Uppsala, Sweden) to remove the organic solvent. Each sample was frozen and dried under vacuum and prepared for liquid chromatography mass spectrometry (LC/MS) or gas chromatography mass spectrometry analysis. Library entries of purified standards or recurrent unknown entities were used to identify compounds. Matches for each sample were verified and corrected as needed.

### 4.5. Statistical Analysis

Student’s *t*-test and one-way ANOVA with post hoc statistical tests were performed using GraphPad Prism statistical software version 6.0. For metabolomics analysis, Welch’s *t*-tests using pairwise comparisons were performed for statistical analysis. Significance for all statistical tests was determined at *p* < 0.05.

## Figures and Tables

**Table 1:** Twenty-five genes with the greatest expression increase in cells treated with WT vs. mock secretomes.

		Mean of Normalized Expression, Duplicates			Expression Ratios				Scaled *eepR*	
Gene symbol	Entrez Gene number	LB control	WT *Serratia*	*eepR* mutant	**WT/cont**	*eepR*/cont	*eepR*/WT	WT/*eepR*	$\frac{\left(eppR-cont\right)}{\left|\left(WT-cont\right)\right|}$	Biological Function
CXCL8 *	3576	41	2346	145	**56.6**	3.5	0.1	16.2	0.04	Inflammatory cytokine
CXCL1 *	2919	51	1452	566	**28.4**	11.1	0.4	2.6	0.37	Inflammatory cytokine
CCL20 *	6364	297	8224	1757	**27.7**	5.9	0.2	4.7	0.18	Inflammatory cytokine
**ITGB8**	3696	4	91	38	**25.7**	10.8	0.4	2.4	0.40	Integrin-mediated cell adhesion
CXCL3	2921	35	830	108	**23.7**	3.1	0.1	7.7	0.09	Chemotaxis
GFPT2	9945	5	96	39	**18.4**	7.4	0.4	2.5	0.37	Glutamine fructose-6-phosphate transaminase
CSF2 *	1437	74	1311	192	**17.7**	2.6	0.1	6.8	0.10	granulocyte macrophage colony-stimulating factor receptor binding
LIF	3976	71	1164	93	**16.4**	1.3	0.1	12.5	0.02	TGF Beta Signaling
CSF3	1440	47	746	146	**16.0**	3.1	0.2	5.1	0.14	granulocyte colony-stimulating factor receptor binding
MMP1 *	4312	104	1639	114	**15.7**	1.1	0.1	14.4	0.01	Proteolysis
**CXCL2**	2920	83	1163	428	**14.1**	5.2	0.4	2.7	0.32	Chemokine
MTSS1	9788	11	137	31	**12.9**	2.9	0.2	4.4	0.16	Actin binding
HCAR3	8843	306	3840	589	**12.6**	1.9	0.2	6.5	0.08	G-protein coupled receptor signaling
IL20	50604	31	379	26	**12.3**	0.8	0.1	14.6	−0.01	Receptor binding
**TNFAIP2**	7127	20	236	198	**11.7**	9.9	0.8	1.2	0.83	Angiogenesis
ICAM1 *	3383	40	445	217	**11.3**	5.5	0.5	2.1	0.44	T cell antigen processing and presentation
**IL36G**	56300	92	1011	536	**11.0**	5.8	0.5	1.9	0.48	Positive regulation of cytokine production
SQSTM1	8878	8	81	28	**10.7**	3.7	0.3	2.9	0.28	Positive regulation of protein phosphorylation
MMP10	4319	100	1064	92	**10.7**	0.9	0.1	11.6	−0.01	Proteolysis
PRDM1	639	139	1394	146	**10.1**	1.1	0.1	9.5	0.01	Negative regulation of transcription from RNA polymerase II
TRAF1	7185	16	160	54	**10.0**	3.4	0.3	3.0	0.27	Apoptosis
**IL1R2**	7850	66	640	412	**9.7**	6.2	0.6	1.6	0.60	Immune response
IL24	11009	474	4413	678	**9.3**	1.4	0.2	6.5	0.05	Apoptosis
MMP9 *	4318	408	3792	2017	**9.3**	4.9	0.5	1.9	0.48	Proteolysis
IL6 *	3569	60	545	152	**9.1**	2.5	0.3	3.6	0.19	Inflammatory cytokine

Seven genes PCR verified (*), see Figure 1. Nine genes in bold also appear in Table 3: “Greatest expression increase in cells treated with *eepR* vs. mock secretomes”.

**Table 2:** Twenty-five genes with the greatest expression decrease in cells treated with WT vs. mock secretomes.

		Mean of Normalized Expression, Duplicates			Expression Ratios				Scaled *eepR*	
Gene Symbol	Entrez Gene number	LB control	WT *Serratia*	*eepR* mutant	**WT/cont**	*eepR*/cont	*eepR*/WT	WT/*eepR*	$\frac{\left(\mathrm{eppR}-\mathrm{cont}\right)}{\left|\left(WT-\mathrm{cont}\right)\right|}$	Biological Function
**TXNIP**	10628	2659	64	320	**0.02**	0.1	5.0	0.2	−0.90	Negative regulation of transcription from RNA polymerase II
**CTGF**	1490	1245	55	35	**0.04**	0.0	0.6	1.6	−1.02	Cartilage condensation
**236865_at**	---	117	7	24	**0.06**	0.2	3.5	0.3	−0.84	Unknown
**ARRDC4**	91947	1338	96	285	**0.07**	0.2	3.0	0.3	−0.85	Positive regulation of ubiquitin-protein ligase activity
LOC100287896	100287896	81	6	38	**0.08**	0.5	5.9	0.2	−0.57	Unknown
NAP1L3	4675	35	4	31	**0.10**	0.9	8.6	0.1	−0.14	Nucleosome assembly
**RP4-813F11.4**	---	146	19	13	**0.13**	0.1	0.7	1.5	−1.05	Unknown
HJURP	55355	747	105	430	**0.14**	0.6	4.1	0.2	−0.49	Nucleosome assembly
PIK3R3	8503	95	14	70	**0.14**	0.7	5.2	0.2	−0.31	Phospholipid metabolic process
SLC26A7	115111	24	4	5	**0.14**	0.2	1.3	0.8	−0.95	Gastric acid secretion
ARRDC3	57561	257	40	113	**0.15**	0.4	2.9	0.4	−0.66	Temperature homeostasis
ZNF750	79755	148	24	62	**0.16**	0.4	2.6	0.4	−0.69	Transcription, DNA-dependent
GPX8	493869	92	15	86	**0.16**	0.9	5.8	0.2	−0.08	Response to oxidative stress
MECOM	2122	154	25	65	**0.16**	0.4	2.6	0.4	−0.69	Neutrophil homeostasis
ENC1	8507	379	64	169	**0.17**	0.4	2.6	0.4	−0.67	Multicellular organismal development
**THAP2**	83591	88	15	11	**0.17**	0.1	0.7	1.4	−1.05	Nucleic acid binding
1560973_a_at	---	34	6	16	**0.18**	0.5	2.7	0.4	−0.63	Unknown
ZNF658	26149	56	10	56	**0.19**	1.0	5.4	0.2	−0.00	Transcription, DNA-dependent
ST6GALNAC5	81849	76	14	44	**0.19**	0.6	3.1	0.3	−0.51	Protein glycosylation
**AOC3**	8639	84	16	8	**0.19**	0.1	0.5	2.0	−1.11	Cell adhesion
AKNAD1	254268	67	13	23	**0.20**	0.3	1.7	0.6	−0.82	Cytoplasm
FAM83D	81610	1588	313	1235	**0.20**	0.8	3.9	0.3	−0.28	Cell cycle
**242708_at**	---	44	9	8	**0.20**	0.2	0.9	1.1	−1.01	Unknown
ZC3H6	376940	99	20	32	**0.21**	0.3	1.6	0.6	−0.85	Nucleic acid binding
* FAM72A	554282	1976	413	1063	**0.21**	0.5	2.6	0.4	−0.58	Cytoplasm

* Full designation of bottom row: FAM72A /// FAM72B /// FAM72C /// FAM72D: Entrez numbers 554282 /// 653820 /// 728833 /// 729533. Eight genes in bold also appear in Table 4: “Greatest expression decrease in cells treated with *eepR* vs. mock secretomes”.

**Table 3:** Twenty-five genes with the greatest expression increase in cells treated with *eepR* vs. mock secretomes.

		Mean of Normalized Expression, Duplicates			Expression Ratios				Scaled *eepR*	
Gene Symbol	Entrez Gene number	LB control	WT *Serratia*	*eepR* mutant	WT/cont	*eepR*/cont	*eepR*/WT	WT/*eepR*	$\frac{\left(\mathrm{eppR}-\mathrm{cont}\right)}{\left|\left(WT-\mathrm{cont}\right)\right|}$	Biological Function
**CXCL1**	2919	51	1452	566	28.4	**11.1**	0.4	2.6	0.37	Inflammatory cytokine
**ITGB8**	3696	4	91	38	25.7	**10.8**	0.4	2.4	0.40	Integrin-mediated cell adhesion
**TNFAIP2**	7127	20	236	198	11.7	**9.9**	0.8	1.2	0.83	Angiogenesis
OLR1	4973	155	1302	1195	8.4	**7.7**	0.9	1.1	0.91	Proteolysis
**IL1R2**	7850	66	640	412	9.7	**6.2**	0.6	1.6	0.60	Immune response
**CCL20**	6364	297	8224	1757	27.7	**5.9**	0.2	4.7	0.18	Inflammatory cytokine
**IL36G**	56300	92	1011	536	11.0	**5.8**	0.5	1.9	0.48	Positive regulation of cytokine production
SLC2A6	11182	34	97	189	2.9	**5.6**	1.9	0.5	2.45	Transport
**ICAM1**	3383	40	445	217	11.3	**5.5**	0.5	2.1	0.44	T cell antigen processing and presentation
**CXCL2**	2920	83	1163	428	14.1	**5.2**	0.4	2.7	0.32	Chemokine
**MMP9**	4318	408	3792	2017	9.3	**4.9**	0.5	1.9	0.48	Proteolysis
CXCL10	3627	115	211	533	1.8	**4.6**	2.5	0.4	4.36	Positive regulation of leukocyte chemotaxis
IL1R2	7850	53	435	241	8.3	**4.6**	0.6	1.8	0.49	Immune response
ICAM1	3383	47	367	213	7.9	**4.6**	0.6	1.7	0.52	T cell antigen processing and presentation
BIRC3	330	27	147	114	5.4	**4.2**	0.8	1.3	0.72	Toll-like receptor signaling pathway
SGPP2	---	51	297	206	5.8	**4.0**	0.7	1.4	0.63	Phospholipid metabolic process
C15orf48	84419	26	92	99	3.5	**3.8**	1.1	0.9	1.11	Mitochondrion
JMJD4	65094	38	56	146	1.5	**3.8**	2.6	0.4	5.88	Protein binding
C6orf132	647024	42	140	159	3.3	**3.8**	1.1	0.9	1.19	Unknown
S100A7	6278	76	168	288	2.2	**3.8**	1.7	0.6	2.31	Response to reactive oxygen species
KMO	8564	27	107	99	3.9	**3.6**	0.9	1.1	0.89	Metabolic process
EFNA1	1942	278	1973	985	7.1	**3.5**	0.5	2.0	0.42	Negative regulation of transcription from RNA polymerase II promoter
FAM20C	56975	148	528	525	3.6	**3.5**	1.0	1.0	0.99	Phosphorylation
**CXCL8**	3576	41	2346	145	56.6	**3.5**	0.1	16.2	0.04	Inflammatory cytokine
KMO	8564	28	121	96	4.4	**3.5**	0.8	1.3	0.74	Metabolic process

Nine genes in bold also appear in Table 1: “Greatest expression increase in cells treated with WT vs. mock secretomes”.

**Table 4:** Twenty-five genes with the greatest expression decrease in cells treated with *eepR* vs. mock secretomes.

		Mean of Normalized Expression, Duplicates			Expression Ratios				Scaled *eepR*	
Gene Symbol	Entrez Gene number	LB control	WT *Serratia*	*eepR* mutant	WT/cont	*eepR*/cont	*eepR*/WT	WT/*eepR*	$\frac{\left(\mathrm{eppR}-\mathrm{cont}\right)}{\left|\left(WT-\mathrm{cont}\right)\right|}$	Biological Function
**CTGF**	1490	1245	55	35	0.04	**0.03**	0.64	1.6	−1.02	Cartilage condensation
**RP4-813F11.4**	---	146	19	13	0.13	**0.09**	0.69	1.5	−1.05	Unknown
**AOC3**	8639	84	16	8	0.19	**0.10**	0.51	2.0	−1.11	Cell adhesion
SERPINE1	5054	81	42	9	0.52	**0.11**	0.22	4.7	−1.86	Regulation of mRNA stability
**TXNIP**	10628	2659	64	320	0.02	**0.12**	5.01	0.2	−0.90	Negative regulation of transcription from RNA polymerase II
**THAP2**	83591	88	15	11	0.17	**0.13**	0.74	1.4	−1.05	Nucleic acid binding
SLC6A13	6540	122	46	18	0.38	**0.15**	0.39	2.6	−1.37	Transport
RFPL3S	10737	31	11	6	0.35	**0.18**	0.53	1.8	−1.25	Unknown
**242708_at**	---	44	9	8	0.20	**0.19**	0.95	1.1	−1.01	Unknown
SLC26A7	115111	24	4	5	0.14	**0.19**	1.31	0.8	−0.95	Gastric acid secretion
EGR3	1960	567	602	108	1.06	**0.19**	0.18	5.6	−12.90	Positive regulation of endothelial cell proliferation
SERTAD4	56256	40	14	8	0.35	**0.20**	0.58	1.8	−1.23	Unknown
**236865_at**	---	117	7	24	0.06	**0.21**	3.55	0.3	−0.84	Unknown
**ARRDC4**	91947	1338	96	285	0.07	**0.21**	2.97	0.3	−0.85	Temperature homeostasis
MYEF2	50804	37	5	8	0.14	**0.23**	1.65	0.6	−0.90	Transcription, DNA-dependent
RYBP	23429	63	31	15	0.49	**0.24**	0.49	2.1	−1.49	Negative regulation of transcription from RNA polymerase II promoter
238548_at	238548_at	44	19	11	0.43	**0.25**	0.59	1.7	−1.31	Unknown
LOC100130705	100130705	67	29	17	0.43	**0.26**	0.60	1.7	−1.30	Unknown
CYR61	3491	5396	1506	1419	0.28	**0.26**	0.94	1.1	−1.02	Regulation of cell growth
ZBTB1	22890	396	217	108	0.55	**0.27**	0.50	2.0	−1.61	Transcription, DNA-dependent
FOS	2353	425	496	117	1.17	**0.28**	0.24	4.2	−4.34	Toll-like receptor signaling pathway
BC034636 /// CTB-113P19.4	---	53	18	15	0.34	**0.28**	0.81	1.2	−1.10	Unknown
ANGPTL4	51129	393	89	111	0.23	**0.28**	1.25	0.8	−0.93	Angiogenesis
UQCRB	7381	47	14	14	0.30	**0.30**	1.02	1.0	−0.99	Oxidative phosphorylation
C1orf52	148423	171	56	52	0.33	**0.30**	0.93	1.1	−1.04	Unknown

Eight genes in bold also appear in Table 2: “Greatest expression decrease in cells treated with WT vs. mock secretomes”.

**Table 5:** Twenty-five genes with the highest scaled *eepR* values (i.e., relatively little effect of WT, relatively large increase by *eepR*).

		Mean of Normalized Expression, Duplicates			Expression Ratios				Scaled *eepR*	
Gene Symbol	Entrez Gene number	LB control	WT *Serratia*	*eepR* mutant	WT/cont	*eepR*/cont	*eepR*/WT	WT/*eepR*	$\frac{\left(\mathrm{eppR}-\mathrm{cont}\right)}{\left|\left(WT-\mathrm{cont}\right)\right|}$	Biological Function
TOMM40L	84134	72	71	237	0.99	3.29	3.33	0.3	235.4	Transport
ARL11	115761	18	19	51	1.01	2.75	2.72	0.4	161.0	Intracellular protein transport
IGFL1	374918	170	173	420	1.02	2.47	2.43	0.4	83.3	Protein binding
227356_at	---	109	112	182	1.02	1.66	1.63	0.6	30.8	Unknown
TMEM177	80775	125	120	221	0.96	1.76	1.84	0.5	18.5	Membrane
TRIM14	9830	221	212	376	0.96	1.70	1.77	0.6	17.8	Protein binding
ZSCAN16	80345	54	52	97	0.95	1.78	1.86	0.5	17.2	Transcription, DNA-dependent
RITA1	84934	80	75	157	0.94	1.97	2.10	0.5	15.1	Intracellular protein transport
KRT34 /// LOC100653049	3885 /// 100653049	202	220	463	1.09	2.29	2.11	0.5	14.8	Epidermis development
CTSC	1075	69	64	135	0.93	1.97	2.11	0.5	13.7	T cell mediated cytotoxicity
FAM13B	51306	128	121	218	0.95	1.70	1.80	0.6	13.1	Signal transduction
CCDC8	83987	70	58	215	0.83	3.08	3.73	0.3	12.1	Negative regulation of phosphatase activity
KIAA1586	57691	34	37	65	1.08	1.91	1.77	0.6	11.9	Nucleic acid binding
COG8 /// PDF	64146 /// 84342	199	217	384	1.09	1.93	1.77	0.6	10.7	Translation
MTRR	4552	418	449	708	1.07	1.69	1.58	0.6	9.4	Sulfur amino acid metabolic process
SLC35F6	54978	125	141	269	1.13	2.15	1.91	0.5	9.1	Establishment of mitotic spindle orientation
CXCL11	6373	121	100	287	0.83	2.38	2.87	0.3	8.1	Positive regulation of leukocyte chemotaxis
HSD17B1	3292	83	103	234	1.24	2.82	2.28	0.4	7.6	Lipid metabolic process
LOC284926	284926	8	13	44	1.63	5.55	3.41	0.3	7.2	Unknown
NOP56	10528	206	233	384	1.13	1.87	1.65	0.6	6.5	rRNA processing
* FAM86B1	*55199	32	26	65	0.82	2.07	2.51	0.4	6.1	Unknown
JMJD4	65094	38	56	146	1.48	3.82	2.58	0.4	5.9	Protein binding
PPAPDC2	403313	67	85	156	1.26	2.33	1.84	0.5	5.0	Metabolic process
AIMP2	7965	1059	966	1505	0.91	1.42	1.56	0.6	4.8	Translation
ZNF165	7718	184	220	358	1.20	1.95	1.63	0.6	4.8	Transcription, DNA-dependent

* full annotation: FAM86B1 /// FAM86B2 /// FAM86C1 /// FAM86DP /// FAM86FP /// FAM86KP: 55199 /// 85002 /// 653113 /// 653333 /// 692099 /// 100287013.

**Table 6:** Twenty-five genes with the lowest scaled *eepR* values (i.e., relatively little effect of WT, relatively large decrease by *eepR).*

		Mean of Normalized Expression, Duplicates			Expression Ratios				Scaled *eepR*	
Gene Symbol	Entrez Gene number	LB control	WT *Serratia*	*eepR* mutant	WT/cont	*eepR*/cont	*eepR*/WT	WT/*eepR*	(*eepR*—cont) |(WT—cont)|	Biological Function
NUFIP2	57532	1790	1786	1204	1.00	0.67	0.67	1.5	−144.7	Protein binding
ZFP36L2	678	3551	3536	2305	1.00	0.65	0.65	1.5	−83.9	Regulation of transcription, DNA dependent
TUFT1	7286	1322	1329	818	1.01	0.62	0.62	1.6	−74.7	Protein binding
PARD6B	84612	495	491	230	0.99	0.46	0.47	2.1	−59.7	Protein complex assembly
GPR157	80045	364	359	208	0.99	0.57	0.58	1.7	−33.0	Signal transduction
ARPC5L	81873	1049	1062	602	1.01	0.57	0.57	1.8	−32.4	Regulation of actin filament polymerization
JUN	3725	1312	1338	621	1.02	0.47	0.46	2.2	−27.1	Angiogenesis
DUSP6	1848	3965	3858	1638	0.97	0.41	0.42	2.4	−21.6	Inactivation of MAPK activity
CD274	29126	373	386	184	1.04	0.49	0.48	2.1	−14.4	Immune response
EGR3	1960	567	602	108	1.06	0.19	0.18	5.6	−12.9	Positive regulation of endothelial cell proliferation
1555897_at	---	89	85	47	0.96	0.53	0.55	1.8	−11.9	Unknown
CHMP1B	57132	220	227	145	1.03	0.66	0.64	1.6	−11.4	Cytokinesis
FHL2	2274	2117	2058	1460	0.97	0.69	0.71	1.4	−11.3	Negative regulation of transcription from RNA polymerase II promoter
E2F7	144455	1501	1411	612	0.94	0.41	0.43	2.3	−10.0	Negative regulation of transcription from RNA polymerase II promoter
SLC2A14 /// SLC2A3	6515 /// 144195	358	342	203	0.96	0.57	0.59	1.7	−9.8	Carbohydrate metabolic process
PHF13	148479	631	661	347	1.05	0.55	0.52	1.9	−9.5	Mitotic cell cycle
JAG1	182	3839	3973	2726	1.03	0.71	0.69	1.5	−8.3	Angiogenesis
SERTAD1	29950	1254	1334	653	1.06	0.52	0.49	2.0	−7.5	Regulation of cyclin-dependent protein serine/threonine kinase activity
KIAA0907	22889	1868	1755	1034	0.94	0.55	0.59	1.7	−7.4	Unknown
SOS1	6654	442	467	268	1.06	0.61	0.58	1.7	−7.1	Apoptotic process
C16orf72	29035	2072	2169	1411	1.05	0.68	0.65	1.5	−6.8	Unknown
RND3	390	1928	1794	1126	0.93	0.58	0.63	1.6	−6.0	GTP catabolic process
SMAD7	4092	271	290	158	1.07	0.58	0.54	1.8	−5.7	Negative regulation of transcription from RNA polymerase II promoter
ADAMTS6	11174	171	187	86	1.10	0.50	0.46	2.2	−5.1	Proteolysis
FZD7	8324	63	60	31	0.89	0.45	0.50	2.0	−5.0	Wnt signaling

**Table 7:** Significantly (*p* < 0.05) enriched canonical pathways which respond to WT *S. marcescens* stimulus.

Canonical Pathway.	−log(*p*-Value)	Number Genes up-Regulated	Number Genes down-Regulated	Total Genes in Pathway
IL-6 Signaling	8.4	20	1	116
Toll-like Receptor Signaling	7.9	16	1	72
NF-kB Signaling	7.0	22	1	164
Colorectal Cancer Metastasis Signaling	5.4	20	1	230
PPAR Signaling	5.0	14	1	90
**TREM1 Signaling**	4.9	12	1	69
HMGB1 Signaling	4.8	15	1	118
Acute Phase Response Signaling	4.6	18	1	166
Role of Pattern Recognition Receptors in Recognition of Bacteria and Viruses	4.2	13	1	118
Cholecystokinin/Gastrin-mediated Signaling	3.3	11	1	99
B Cell Activating Factor Signaling	3.1	7	1	40
**LXR/RXR Activation**	3.1	12	1	120
Pancreatic Adenocarcinoma Signaling	3.0	10	1	106
Glioma Invasiveness Signaling	2.8	7	1	57
Cell Cycle: G2/M DNA Damage Checkpoint Regulation	2.6	1	1	49
NF-kB Activation by Viruses	2.1	7	1	73
NRF2-mediated Oxidative Stress Response	2.0	12	1	175
CXCL8 Signaling	1.9	13	1	
Tec Kinase Signaling	1.8	10	2	183
MIF Regulation of Innate Immunity	1.8	5	0	150
iNOS Signaling	1.6	5	0	39
Antioxidant Action of Vitamin C	1.6	8	0	43
PPARα/RXRα Activation	1.5	12	0	91
Phospholipase C Signaling	1.3	13	1	165

The two pathways in the bold text were also significantly stimulated by *eepR* secretomes with the same −log(*p* values) found for WT secretomes. Note: This indicates that most immune pathways in this table modulated by WT secretome treatment are not modulated by *eepR* secretome treatment.

## Data Availability

Microarray data was deposited to NCBI gene expression Omnibus (GEO accession number GSM1832614). Metabolomic data is supplied in Table S2.

## Supplementary Materials

The following are available online at https://www.mdpi.com/article/10.3390/antibiotics10070770/s1, Table S1: Metabolomics analysis of WT and *eepR* secretome-treated HCLE, Table S2: Metabolomics data.
